# The Causes and Consequences of Transient Epileptic Amnesia

**DOI:** 10.3233/BEN-2011-0340

**Published:** 2011-11-07

**Authors:** Christopher R. Butler, Adam Zeman

**Affiliations:** ^1^Department of Clinical NeurologyUniversity of OxfordOxfordUK; ^2^Department of NeurologyPeninsula Medical SchoolUniversity of ExeterUK

## Abstract

Transient epileptic amnesia (TEA) is a recently recognised syndrome of epilepsy in which the principle manifestation of seizures is recurrent episodes of isolated memory loss. In this article, we describe the clinical and cognitive profile of this emerging syndrome, and present new data that provide at most weak support for its proposed relationship to cerebrovascular disease. TEA is often associated with two unusual forms of interictal memory impairment: accelerated long-term forgetting and remote memory impairment. We discuss the clinical and theoretical implications of these relatively novel cognitive deficits.

## 1. Introduction

Transient amnesia is a striking clinical phenomenon. However, in the majority of cases its cause remains a mystery. Over recent years, it has been recognised that a proportion of patients who experience recurrent episodes of isolated memory loss have a form of epilepsy that has been called transient epileptic amnesia (TEA). In this article, we describe the clinical and cognitive profile of this emerging syndrome, including new data that provide at most weak support for its proposed relationship to cerebrovascular disease. We also discuss the clinical and theoretical implications of the unusual forms of interictal memory impairment that often accompany TEA.

## 2. TEA: development of the concept

Perhaps the earliest description of epilepsy-related transient amnesia is to be found in Hughlings-Jackson's report [1] of Dr. Z, a physician with focal epilepsy who, whilst at work, experienced the onset of his typical epileptic aura. He subsequently examined, diagnosed and treated a child with pneumonia, yet later had no recollection of the consultation. Some years later, Z's brain came to autopsy, and a single, circumscribed lesion in the left uncus was discovered [2].

Over subsequent decades, a steady stream of case reports and short series of patients with epileptic amnesia appeared in the literature. These are reviewed in Butler and Zeman [3]. The term 'transient epileptic amnesia' was introduced by Kapur [4,5], who highlighted that amnesic attacks caused by epilepsy are similar to the well-recognised syndrome of transient global amnesia (TGA), but may have certain distinguishing features, including brevity and recurrence. Zeman and colleagues reported the clinical profile of 10 patients with TEA and proposed diagnostic criteria (see below) [6]. Most recently, Butler and colleagues have described the clinical, neuropsychological and radiological features of 50 patients with TEA [7], as part of the UK-wide TIME (The Impairment of Memory in Epilepsy) Project.

The diagnostic criteria for TEA are as follows:

1. A history of recurrent witnessed episodes of transient amnesia
2. Cognitive functions other than memory judged to be intact during typical episodes by a reliable witness
3. Evidence for a diagnosis of epilepsy based on one or more of the following:
   a. epileptiform abnormalities on electroencephalography

ISSN 0953-4180/11/$27.50 © 2011 – IOS Press and the authors. All rights reserved b. the concurrent onset of other clinical features of epilepsy (e.g. lip-smacking, olfactory hallucinations)
c. a clear-cut response to anticonvulsant therapy.

## 3. Clinical features of TEA

The amnesic attacks of TEA typically begin in late middle age, with a mean age of onset of 57 years. Two thirds of patients are male. The episodes of amnesia are generally briefer that those of TGA, lasting a median of 30 to 60 minutes, although longer episodes are not uncommon. As with most forms of epilepsy, the frequency of attacks is highly variable but, on average, they occur about once per month. In contrast, TGA recurs at a rate of only 6–10% per year [8]. Another helpful clue to the diagnosis is that episodes of TEA characteristically occur upon waking, with around 70% of patients experiencing at least some attacks in this context. During the amnesic episode, patients have difficulty laying down new memories (anterograde amnesia) and retrieving memories for past events (retrograde amnesia). The anterograde component is often partial: 44% of patients later say they can “remember not having been able to remember”.

Whilst amnesia is the predominant feature of TEA attacks, careful enquiry can, in some instances, reveal other signs suggestive of epilepsy. The most common of these is olfactory or gustatory hallucinosis, experienced by up to 50% of patients with TEA. Subtle oral automatisms (lip smacking or chewing) or brief periods of unresponsiveness may also accompany some attacks. A few patients develop more clear-cut ‘complex partial seizures’, but generalised tonic-clonic convulsions are rare. Interictal EEG reveals epileptiform abnormalities in only about one third of cases, although sensitivity may be significantly enhanced by sleep-deprivation. Magnetic resonance imaging (MRI) of the brain is usually clinically unremarkable. The amnesic attacks of TEA typically respond well to low dose monotherapy with an antiepileptic drug. However, treatment is often delayed as patients may be misdiagnosed as having TGA, ‘psychogenic attacks’ or dementia [7].

Despite effective treatment of the amnesic episodes, 81% of patients with TEA complain of significant persistent – i.e. interictal – memory difficulties [3]. The problems they describe are unusual. Of the 50 TEA patients in the TIME Project, i) 70% reported loss of memories for salient, personally experienced events from the remote past; ii) 44% described the excessively rapid fading of newly acquired memories over a period of days to weeks; iii) 36% reported new difficulties with spatial navigation, even around previously familiar environments. These atypical memory deficits are discussed in more detail below.

## 4. Pathophysiology of TEA

Several clinical features of TEA – the mixed anterograde and retrograde amnesia, associated olfactory hallucinations and the localisation of epileptiform abnormalities on scalp EEG – point to the medial temporal lobes as being the seizure source. In one case, FDG-PET imaging during a prolonged episode of ictal amnesia revealed isolated hypermetabolism in the left hippocampus, a finding that had resolved three months later [9]. Furthermore, the series of 50 TEA patients studied by Butler et al. [7] had localised but subtle volume loss in the medial temporal lobes bilaterally.

Ten published case reports of TEA have included EEG data gathered during an amnesic attack [3]. Of these, six demonstrated the amnesia as an ictal phenomenon, whereas in four it was found to be postictal. Ictal epileptiform EEG changes were bilateral in eight of the ten patients. Interictal epileptiform abnormalities, when present, are also more often than not bilateral – 56% bilateral, 32% left-sided, 12% right-sided [3].

## 5. Aetiology of TEA

Focal brain lesions, always in the temporal region, have been reported in a small minority of TEA cases and include tumours (meningioma [10], glioblastoma multiforme [11], metastasis [12]), haemosiderin deposition [13] and hippocampal sclerosis [9]. Occasionally, transient episodes of dense amnesia and wandering may occur as a result of seizures in patients with neurodegenerative dementia [14]. However, in most cases of TEA, no clear cause for the epilepsy is identified and, as noted above, brain imaging is usually unremarkable. There is no evidence of an increased prevalence of recognised risk factors for epilepsy such as birth injury, febrile seizures, head injury, intracranial infection or family history of epilepsy [7]. Zeman and colleagues [6] noticed a high prevalence of cardiac disease amongst the ten patients described in their series and postulated a role for cerebrovascular disease in the aetiology of TEA. We investigated this possibility in the 50 patients from the TIME Project by quantifying risk factors and radiological signs of cerebrovascular disease.

### 5.1. Cerebrovascular disease risk factors

Using a series of standard questions, data were collected from patients regarding: 1) previous events potentially associated with cerebral hypoxia including cardiac arrhythmias, cardiac valve disease, myocardial infarction or other possible hypotensive episodes and 2) cerebrovascular disease risk factors (CVRFs) comprising hypertension, diabetes mellitus, smoking, previous TIA or stroke, ischaemic heart disease or peripheral vascular disease and any family history of stroke, ischaemic heart disease or peripheral vascular disease.

Control data were obtained from the Lothian Birth Cohort 1936 Study (LBC1936). This ongoing study, run by the University of Edinburgh Department of Psychology, collects clinical and neuropsychological data from people who were born in 1936 and underwent cognitive testing at the age of 11 as part of the "Scottish Mental Survey". This healthy control dataset was chosen because of its large size and mean age of 67 years, close to that of the TIME patients. In clinical studies, CVRFs tend to have a relatively small effect on the outcomes of interest so having a large sample size is critical. For the purposes of the TIME Project, participant records from LBC1936 were examined and information about CVRFs was extracted using a pro forma identical to that used for TEA patients. Information about hyperlipidaemia in the control cohort was unfortunately not available and could therefore not be included in the analysis.

The cerebrovascular risk factor data are shown in Table 1. The patient and control groups were well matched for age although the variance differed (patients: mean = 68.3 years (SD = 8.6), controls: mean = 67.0 years (SD = 1.1), $p = 0.320$). The proportion of males was greater in the patient group (odds ratio (OR) = 2.08, 95% confidence interval (CI) = 1.11 to 3.90, $p = 0.02$). The CVRFs significantly overrepresented in the patient group were: cardiac arrhythmias (OR = 4.62, CI = 1.63 to 13.12, $p = 0.008$), cardiac valve disease (OR = 4.09, CI = 1.18 to 14.10, $p = 0.038$) and arterial aneurysm (OR = 15.96, CI = 1.4 to 179.31, $p = 0.003$). Of the six TEA patients with a history of cardiac arrhythmia, four had paroxysmal atrial fibrillation and two had been diagnosed with intermittent complete heart block following collapses and fitted with a permanent pacemaker. Of the four patients with cardiac valve disease, two had mild aortic stenosis, one aortic incompetence, and one had had an aortic valve replacement. Two patients and one control subject had undergone elective repair of an abdominal aortic aneurysm. The number of patients in these CVRF categories was low and the lower limit of the confidence intervals was close to 1 in each case. Cigarette smoking was underrepresented in the patient group (OR = 0.32, CI = 0.20 to 0.65, $p < 0.001$). There was therefore no thoroughly compelling evidence for an increased prevalence of CVRFs amongst TEA patients.

### 5.2. Radiological markers of cerebrovascular disease

Besides revealing previous infarction and haemorrhagic stroke, MRI can disclose signs of covert small-vessel disease. White matter hyperintensities (WMH), seen on T2-weighted and fluid-attenuated inversion recovery (FLAIR) MRI sequences, represent regions of scattered white matter loss with increased tissue water content. They are common in older adults and correspond to ischaemia from hypoperfusion and small-vessel disease [15]. Their extent correlates with certain cerebrovascular risk factors including hypertension [16] and diabetes [17]. Moreover, their presence is associated with cognitive decline [18,19]. These imaging abnormalities may, therefore, provide a robust objective indication of the presence and extent of small-vessel disease.

The 50 TEA patients in the TIME Project all underwent MR brain imaging as described in Butler et al. [7]. Control imaging data were obtained from 70 healthy, age-matched control subjects. Axial T2-weighted and FLAIR images were rated for WMH using the Fazekas scale [20]. This widely used scale distinguishes between periventricular and deep white matter hyperintensities.

The WMH data are shown in Table 2. Patients and controls were well matched for age (patient mean = 68.2 (SD = 8.8), control mean = 68.5 (SD = 5.1), $p = 0.859$) and sex (patients: 31 males; controls: 48 males; $\chi 2 = 0.001$; $p = 0.976$). For comparison of proportions, the datasets were dichotomised into mild (WMH score 0 or 1) or moderate to severe (WMH score 2 or 3). WMH scores were not significantly different between the patient and control group for either periventricular WMH ($\chi 2 = 1.195$; $p = 0.274$) or deep WMH ($\chi 2 = 0.345$, $p = 0.557$).

While the elevated levels of cardiac dysrrhythmia and valve disease may provide a clue, the aetiology of TEA remains unclear. These new data do not support the hypothesis that TEA is caused by cerebrovascular disease. However, it is important to note the possibility that subtle but strategically located vascular lesions may act as a seizure focus but not be detectable with

Table 1
Cerebrovascular disease risk factors in TEA patients and LBC1936 healthy control subjects

| | Patients ($n$ = 50) | Controls ($n$ = 434) | Odds ratio | 95% confidence interval | p |
|---|---|---|---|---|---|
| mean age (SD) (yrs) | 67.0 (8.7) | 67.0 (1.1) | n/a | n/a | n/a |
| sex (M/F) | 34 /16 | 194 /190 | 2.08 | 1.11–3.90 | 0.020 |
| previous TIA/stroke | 3 (6%) | 13 (3%) | 1.82 | 0.50–6.63 | 0.276† |
| hypertension | 23 (46%) | 135 (31%) | 1.57 | 0.87–2.85 | 0.134 |
| diabetes mellitus | 2 (4%) | 33 (8%) | 0.43 | 0.13–1.91 | 0.204† |
| IHD (MI or angina) | 6 (12%) | 54 (12%) | 0.83 | 0.34–2.05 | 0.691 |
| PVD | 1 (2%) | 4 (1%) | 1.94 | 0.21–17.70 | 0.459† |
| cardiac arrhythmia | 6 (12%) | 11 (3%) | 4.62 | 1.63–13.12 | 0.008† |
| cardiac valve disease | 4 (8%) | 8 (2%) | 4.09 | 1.18–14.10 | 0.038† |
| arterial aneurysm | 2 (4%) | 1 (< 1%) | 15.96 | 1.4–179.31 | 0.003† |
| cigarette smoking (ever) | 13 (26%) | 202 (47%) | 0.32 | 0.20–0.65 | < 0.001 |
| alcohol excess | 9 (18%) | 62 (14%) | 1.14 | 0.53–2.47 | 0.739 |
| FHx of TIA/stroke/IHD/PVD | 17 (34%) | 157 (36%) | 0.75 | 0.40–1.38 | 0.35 |

†Fisher's Exact Test.
FHx = family history; IHD = ischaemic heart disease; MI = myocardial infarction; PVD = peripheral vascular disease; TIA = transient ischaemic attack.

the imaging techniques used in this study. Alternative possible aetiologies include neurodegenerative disease or immune-mediated limbic encephalitis. A recent single case study [21] reported an improvement in neuropsychological profile after a two year period of treatment with anticonvulsant medication, leading the authors to argue against a neurodegenerative aetiology in this patient. Further studies with more sensitive imaging, longitudinal follow-up, autoantibody measurement and pathological examination are needed.

Table 2
White matter hyperintensities in TEA patients and healthy controls

| | Score | Patients ($n$ = 47) | Controls ($n$ = 70) |
|---|---|---|---|
| Age | | 68.2 (8.8) | 68.5 (5.1) |
| Sex (M/F) | | 31 /16 | 48 /22 |
| White matter hyperintensities | | | |
| PVH | 0 | 5 (10%) | 2 (3%) |
| | 1 | 22 (46%) | 31 (44%) |
| | 2 | 15 (32%) | 33 (47%) |
| | 3 | 5 (12%) | 4 (6%) |
| DMWH | 0 | 7 (15%) | 4 (6%) |
| | 1 | 36 (76%) | 62 (89%) |
| | 2 | 1 (2%) | 4 (6%) |
| | 3 | 3 (7%) | 0 (0%) |

PVH = periventricular hyperintensities, DWMH = deep white matter hyperintensities.

## 6. Neuropsychology of TEA

Despite the widespread complaints of poor memory amongst patients with TEA, performance on standard neuropsychological tests is usually intact. In the 50 patients described by Butler et al. [7], scores on tests of general intelligence, language, executive function and visuospatial perception were not significantly different from closely matched controls. Tests of anterograde memory did reveal a subtle decrement in performance across the group (about 1 standard deviation below the control group mean), and scores were positively correlated with the volume of medial temporal lobe structures [22]. Nevertheless, the patients' performance on these tests did not correlate with their subjective memory complaints and was well within the range considered to be clinically normal. The discrepancy between subjective and objective memory performance may, in part, reflect the limited scope of standard neuropsychological tests which, for understandable reasons, do not examine memory retention over extended delays of more than about 30 minutes, and rarely probe autobiographical recollection. However, as we discuss in the following sections, accelerated long-term forgetting and remote memory impairment are widespread in TEA, have also been demonstrated in other forms of epilepsy, and offer new arenas to explore the underpinnings of human memory function.

## 7. Accelerated long-term forgetting

Approximately 50% of patients with TEA describe the excessively rapid fading of newly acquired memories over a period of days to weeks [7]. For example, a university professor visited Italy for a conference and, on his return, presented a slide show of the trip, with a detailed narrative, to his family. However, 1 month later he had no recollection of the trip at all [9]. Pa-

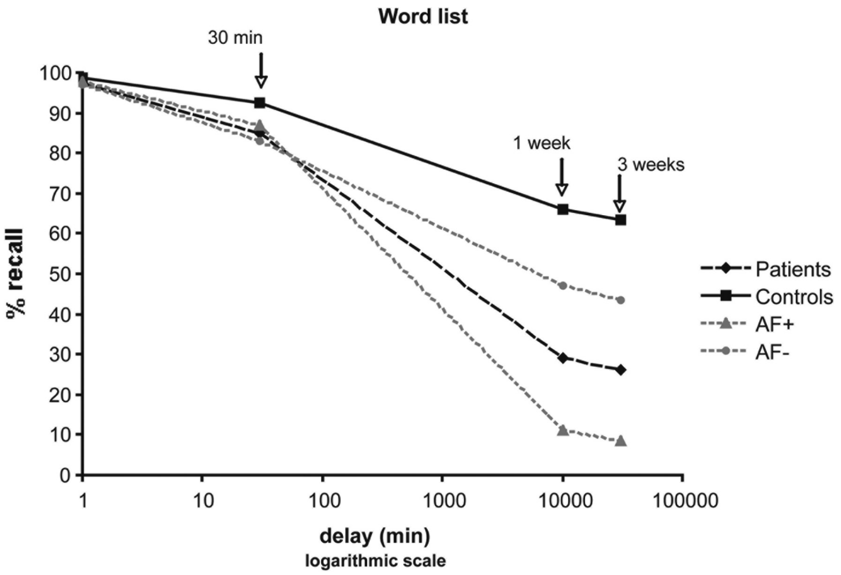

Fig. 1. Accelerated long-term forgetting in a group of 24 patients with TEA, 12 of whom complained specifically of accelerated long-term forgetting (AF+), whereas 12 did not (AF−).

tients with TEA show normal learning of a word list, and normal recollection after a 30 minute delay. However, at longer delays, memory for the words is dramatically impaired (see Fig. 1). This accelerated long-term forgetting (ALF) has also been demonstrated for non-verbal material [7,23] as well as events outside the laboratory [24], and correlates much better with subjective memory complaints than does performance on standard memory tests [22]. Recent evidence [24] suggests that the critical period of forgetting may be the first 24 hours after learning, although this is likely to vary from patient to patient.

The cause of ALF remains unknown. Given the apparently normal learning and initial retention of information by these patients, a compelling interpretation of the deficit is that it reflects disturbed memory consolidation. In healthy subjects, the consolidation of newly acquired memories is thought to involve gradual reorganization of the memory trace so that it becomes independent of the hippocampus. In TEA, no relationship has been found between long-term forgetting and the volume of medial temporal or other brain regions [22]. It has therefore been suggested that the disruption of memory consolidation is instead related to (subclinical) seizure activity. This suggestion gains some support from a few case studies demonstrating improvement of long term memory after the initiation of antiepileptic medication [25,26].

ALF has been reported in association with other forms of epilepsy [27–30] (see [3,31] for reviews). It is a clinically important phenomenon meriting continuing investigation into its causes and response to treatment. ALF is also likely to afford new insights into processes of long-term memory consolidation. For example, the observation that TEA attacks often occur upon waking, alongside mounting evidence that memory consolidation is critically dependent upon post-learning sleep [32], raises the intriguing possibility that ALF reflects impairment of sleep-dependent consolidation by nocturnal epilepsy-related neural activity.

## 8. Remote memory impairment

Up to 70% of patients with TEA complain of a patchy loss of autobiographical memories, affecting the recollection of salient events from their remote past, events that occurred even decades prior to the onset of seizures [7]. Many report that these memories do not resurface even with extensive verbal or photographic cuing. Recent work [33] has revealed severe deficits in the retrieval of episodic autobiographical memories from across the lifespan. In contrast, memory for public events was affected to a much milder degree and only for events of the most recent decade. Some TEA patients – around one third – also describe new difficulties with recognizing previously familiar places and navigating familiar routes.

As with ALF, the cause of remote memory impairment in TEA is unknown. The lack of response to cuing, and the apparent patchiness of the memory loss, suggests a problem with the storage of memory traces, rather than a general impairment of retrieval mechanisms. However, we have recently encountered a patient in whom long lost memories were recovered following a series of episodes of déjà vu [34], indicating that in some cases the deficit may indeed lie at the level of retrieval. No relationship has been found between the extent of autobiographical memory impairment and regional brain volumes in TEA [22]. Nevertheless, the possibility remains that subtle structural damage, perhaps in the medial temporal lobes but beyond the resolution of imaging techniques used to date, is responsible. An alternative explanation is that epileptic activity, spreading through the autobiographical memory network, somehow causes irreparable damage to established memory traces. Whatever the underlying mechanism, it is not immediately clear that current neuroscientific models of memory can account for such an extensive loss of remote autobiographical memory in the context of relatively preserved new learning (see [3] for further discussion).

## 9. Borderlands of TEA

As we have discussed in this article, the syndrome of TEA is characterised by recurrent episodes of isolated amnesia that begin in middle to old age, often occur upon waking, typically last about 30 minutes and respond well to anticonvulsant medication. Patients are frequently misdiagnosed as having transient global amnesia. The amnesic attacks often go hand in hand with persistent but difficult to measure cognitive deficits including ALF and remote memory impairment. As with any syndrome, TEA has somewhat indistinct boundaries. A notable example is the overlap with what Gallassi and colleagues have termed the Epileptic Amnesic Syndrome (EAS) [35,36]. Patients with EAS have late onset (mean age = 63 years) persistent memory difficulties, often detectable on standard neuropsychological instruments, in association with subtle temporal lobe seizures. In other patients (e.g. [37]), complaints of ALF and remote memory impairment can precede the onset of amnesic seizures by several years. Further research is necessary to clarify the relationship between TEA and other forms of late-onset temporal lobe epilepsy. In the meantime, the concept serves to highlight this distinctive presentation of epilepsy, and facilitate novel explorations of human memory function.

## Acknowledgements

The authors are grateful to Dr J Penge for assistance with gathering data on cerebrovascular disease risk factors and white matter hyperintensities on brain imaging. We also thank Professor Ian Deary for permitting our use of data from the LBC1936 study.
